# Physiological assessment of left ventricular size indexed by peak oxygen uptake across sporting disciplines

**DOI:** 10.1093/ehjimp/qyaf138

**Published:** 2025-10-30

**Authors:** Jana Schellenberg, Lynn Matits, Johannes Kersten, Daniel Alexander Bizjak, Johannes Kirsten, Thomas Fremo, Arnt Erik Tjønna, Knut Skovereng, Øyvind Sandbakk, Inger-Lise Aamot Aksetøy, Knut Asbjørn Rise Langlo, Håvard Dalen, Jon Magne Letnes

**Affiliations:** Sports and Rehabilitation Medicine, University Hospital Ulm, Leimgrubenweg 14, Ulm 89075, Germany; Sports and Rehabilitation Medicine, University Hospital Ulm, Leimgrubenweg 14, Ulm 89075, Germany; Clinical & Biological Psychology, Institute of Psychology and Education, Ulm University, Ulm 89075, Germany; Sports and Rehabilitation Medicine, University Hospital Ulm, Leimgrubenweg 14, Ulm 89075, Germany; Sports and Rehabilitation Medicine, University Hospital Ulm, Leimgrubenweg 14, Ulm 89075, Germany; Sports and Rehabilitation Medicine, University Hospital Ulm, Leimgrubenweg 14, Ulm 89075, Germany; Department of Circulation and Medical Imaging, Norwegian University of Science and Technology, Prinsesse Kristinas gate 3, Trondheim 7030, Norway; Department of Circulation and Medical Imaging, Norwegian University of Science and Technology, Prinsesse Kristinas gate 3, Trondheim 7030, Norway; Centre for Elite Sports Research, Department of Neuromedicine and Movement Science, Norwegian University of Science and Technology, Trondheim 7491, Norway; School of Sport Science, UiT the Arctic University of Norway, Tromsø, Norway; Department of Circulation and Medical Imaging, Norwegian University of Science and Technology, Prinsesse Kristinas gate 3, Trondheim 7030, Norway; National Advisory Unit on Exercise Training as Medicine for Cardiopulmonary Conditions, Prinsesse Kristinas gate 3, Trondheim 7030, Norway; Clinic of Rehabilitation, St. Olavs University Hospital, Trondheim, Norway; Department of Nephrology, Clinic of Medicine, St. Olavs Hospital, Trondheim University Hospital, Prinsesse Kristinas gate 3, Trondheim 7030, Norway; Department of Clinical and Molecular Medicine, Norwegian University of Science and Technology, Trondheim, Norway; Department of Circulation and Medical Imaging, Norwegian University of Science and Technology, Prinsesse Kristinas gate 3, Trondheim 7030, Norway; Department of Medicine, Levanger Hospital, Nord-Trøndelag Hospital Trust, Kirkegata 2, Levanger 7600, Norway; Clinic of Cardiology, St. Olavs University Hospital, Prinsesse Kristinas gate 3, Trondheim 7030, Norway; Department of Circulation and Medical Imaging, Norwegian University of Science and Technology, Prinsesse Kristinas gate 3, Trondheim 7030, Norway; Clinic of Cardiology, St. Olavs University Hospital, Prinsesse Kristinas gate 3, Trondheim 7030, Norway

**Keywords:** cardiac remodelling, left ventricular size, peak oxygen uptake, athlete’s heart, sports cardiology, training adaptations, cardiopulmonary exercise testing

## Abstract

**Aims:**

Left ventricular (LV) enlargement is a common training-induced adaptation in athletes, particularly in endurance sports. Previous research indicates that indexing LV volumes and mass to absolute peak oxygen uptake (VO₂_peak_) better reflects physiological adaptation than traditional indexing to body surface area (BSA). Therefore, we investigated whether indexing LV end-diastolic volume (LVEDV) and mass to VO_2peak_ could eliminate differences in LV size among athletes from different sport categories (endurance, mixed, power, and technical).

**Methods and results:**

This analysis included 70 athletes from the multicenter COSMO-S in Germany and 15 elite endurance athletes from Norway. All participants (29 ± 8 years, 52 male) underwent echocardiography and cardiopulmonary exercise testing. In regression analyses, VO_2peak_ (L/min) accounted for a significantly greater proportion of the variance in LVEDV than BSA (R^2^ 0.64 vs. 0.19, *P* < 0.001), while this difference was not significant for LV mass (R^2^ 0.54 vs. 0.36, *P* = 0.06). When indexed to BSA, both LVEDV and LV mass revealed significant differences across sports (both *P* ≤ 0.019), that disappeared when indexed to VO₂_peak_ (all *P* ≥ 0.40). In a cohort of 12 dilated cardiomyopathy (DCM) patients serving as a pathological reference group, indexing LVEDV and LV mass to VO_2peak_ better differentiated DCM patients from athletes than indexing to BSA.

**Conclusion:**

Indexing LV size to VO₂_peak_ may provide a more physiological interpretation of cardiac adaptations in athletes and reduce sport-specific differences due to better consideration of training-induced adaptations. These findings should be replicated in larger cohorts and tested for the ability to detect subtle pathologies.

## Graphical Abstract

**Graphical Abstract:**
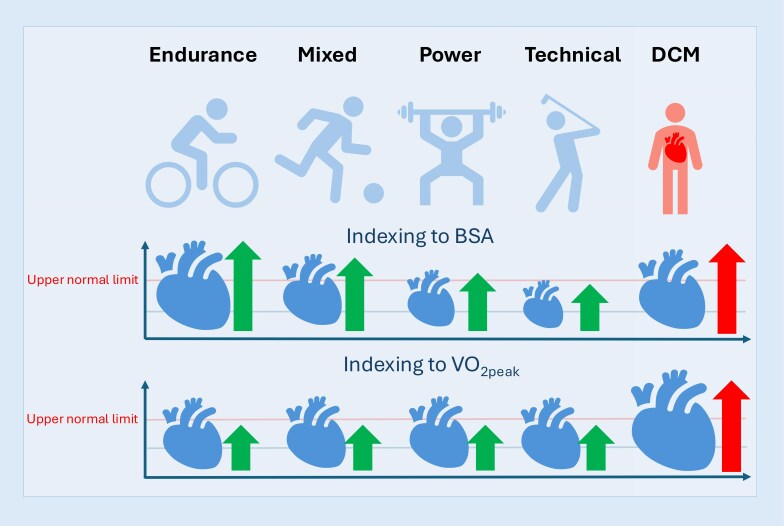
Indexing LV volume and mass to VO_2peak_ removes differences across sporting disciplines and improves discrimination between athletes and patients with dilated cardiomyopathy.

## Introduction

Left ventricular (LV) enlargement is relatively common in athletic individuals, particularly those engaged in endurance training.^1^ It has been proposed that beyond age, sex, ethnicity and body size, the impact of the specific sporting discipline should be considered in the assessment of the athlete’s heart.^2^ Sporting disciplines have been categorized into technical, power, mixed and endurance to distinguish cardiovascular adaptations associated with each type of sport.^3^ Accordingly, reference ranges for cardiac structure specific to different sporting disciplines have been developed.^4^ However, these ranges are inconsistently defined, not always available, and may complicate rather than simplify diagnostic decision-making in clinical practice. A novel approach to evaluating cardiac size and to determine if cardiac chambers are physiologically or pathologically enlarged, has recently been proposed, with LV end-diastolic volume (EDV) being indexed to absolute peak oxygen uptake (VO_2peak_, L/min).^5^ The method leverages a strong, well-established relationship between these factors in healthy individuals.^5,6^ In previous work, this approach was able to distinguish cardiac enlargement due to heart failure from that due to athletic training, unlike indexing by body surface area (BSA), which can be misleading in athletic populations because it does not account for variations in training-induced adaptations. While a previous study established VO_₂peak_-based indexing as a promising approach, the present study builds on this concept by examining whether such indexing eliminates differences across endurance, mixed, power, and technical athletes, and by testing its potential clinical utility for distinguishing physiological from pathological enlargement using a reference group of patients with dilated cardiomyopathy. The inclusion of athletes from diverse sporting disciplines reflects the heterogeneity of real-world athletic populations seen in clinical practice and allows evaluation of whether VO_₂peak_-indexing remains consistent across a broad spectrum of training adaptations. From a clinical perspective, VO_₂peak_-indexing could reduce reliance on sport-specific reference ranges, which are cumbersome to apply, inconsistently validated, and often lack external validation, offering instead a more practical and broadly applicable tool for clinical decision-making. Therefore, the primary aim of this study was to determine whether indexing LVEDV to VO_2peak_ could reduce or eliminate the need for sport-specific reference ranges for cardiac chamber size. We hypothesized that LVEDV and LV mass indexed to BSA would differ significantly across sporting disciplines due to their different cardiovascular demands, whereas indexing to VO_2peak_ would eliminate the variation between sports. Additionally, we hypothesized that indexing LVEDV and LV mass to VO_2peak_ would better differentiate pathological from physiological adaptations.

## Methods

### Study population

This study included data on elite and recreational athletes from two cohorts. The first cohort derived from the prospective *COVID-19 in Elite Sports* – a multicenter cohort study (COSMO-S),^7^ which enrolled elite and recreational athletes aged >18 years across various sports (endurance, mixed, power, or technical)^3^ who had tested positive for SARS-CoV-2 via PCR or had positive antibodies with typical COVID-19 symptoms. The present work is a secondary, retrospective, cross-sectional analysis of prospectively collected data from COSMO-S and a Norwegian elite athlete cohort. The study hypothesis was formed prior to data analysis. For this analysis, only data collected at least three months after acute infection were included, and all athletes were free of signs of active infection or persistent symptoms at the time of assessment. However, residual cardiopulmonary effects related to prior infection cannot be entirely excluded. The Norwegian cohort comprised elite endurance athletes competing at national, international and Olympic levels, evaluated as a part of their regular follow-up at the Centre for Elite Sports Research in Trondheim, Norway. In COSMO-S, all athletes defined as elite trained >10 h weekly and competed professionally. Recreational athletes trained at least three times per week, totalling >20 metabolic equivalents of task and five to eight hours of exercise weekly. All participants provided written informed consent. Both cohorts have been described comprehensively in previous publications.^7–9^ For this study, athletes above 50 years and/or a resting LV ejection fraction ≤45% were excluded.

In addition, we included a reference group of patients with dilated cardiomyopathy (DCM) from a randomized clinical trial conducted in Trondheim, Norway. For the present study, we report baseline measurements performed prior to any interventions in that trial.^10^

The study adhered to the Declaration of Helsinki and was approved by the local ethics committees (EK 408/20, REC mid-Norway 13 083/7160/15409).

### Clinical evaluation

All participants underwent comprehensive clinical evaluations, which included medical history, physical examination, blood sampling, calculation of BSA and body mass index (BMI), a 12-lead electrocardiogram (ECG), resting two-dimensional (2D) transthoracic echocardiography, and cardiopulmonary exercise testing (CPET).

### Transthoracic echocardiography

Transthoracic echocardiography was performed by experienced operators using an EPIQ 7 ultrasound system with a phased-array probe X5-1 (Philips GmbH, Hamburg, Germany) in COSMO-S, and a Vivid E95 scanner with a 4Vc-D matrix transducer (GE Vingmed Ultrasound, Horten, Norway) for the Trondheim cohort, adhering to current guidelines.^11^ Analyses were performed locally at each study centre. Simpsons biplane method was used for assessment of LVEDV, LV end-systolic volume, and LVEF. LV internal diameters (LVIDd), interventricular septal thickness (IVSd) and posterior wall thickness (PWd) at end-diastole were measured from parasternal long-axis views in 2D images. LV mass was calculated using the linear method.^11^ Tricuspid annular plane systolic excursion (TAPSE) was measured in the focused four-chamber view using M-mode. Diastolic function was characterized by the E/A and E/e´ ratio using the mean of lateral and septal e´ in the calculation.^12^

### Cardiopulmonary exercise testing

In the German cohort, cardiopulmonary exercise testing was conducted on a bicycle ergometer (Excalibur Sport, LODE B.V., Groningen, The Netherlands) with a breath-by-breath gas analysis system (Ergostik, Geratherm Respiratory, Bad Kissingen, Germany). In the Trondheim cohort, testing was performed using a mixing-chamber Vyntus CPX system (ErichJaeger GmbH, Hoechberg, Germany) and the sport-specific modality assigned to the athlete (bicycle ergometer, treadmill or roller ski treadmill).

In COSMO-S, all athletes completed an incremental ramp protocol tailored to age, gender, body mass and fitness level, aiming for exhaustion within 8–12 min.^13^ In Trondheim, after a warm-up, skiers and runners performed an incremental protocol with a 1.0 km/h increase per minute, while cyclists underwent a 25 W increase per minute, with individualized starting points designed to reach exhaustion within approximately 10 min. The DCM patients performed an individualized protocol, walking on a treadmill at constant speed with increasing inclination every other minute until voluntary exhaustion, using the same Vyntus CPX system (Erich Jaeger GmbH, Hoechberg, Germany) as the athletes in Trondheim. The evaluation followed established guidelines.^14^ Key parameters analysed included VO_2peak_, heart rate (HR) at rest and at VO_2peak_, and peak respiratory exchange rate (RER). Unless otherwise specified, VO_2peak_ refers to the absolute VO_2peak_ throughout this paper.

### Statistical analysis

Descriptive data are presented as mean ± SD, and as numbers and percentages. Percentage of predicted VO_2peak_ was calculated by the FRIEND equation as (measured/predicted) * 100.^15^ Due to small sample sizes in some sport types, the assumption of normality was violated. Therefore, a Kruskall-Wallis H test was used to assess differences in LVEDV, LV mass indexed to BSA, and VO_2peak_ across different sporting disciplines. We also performed multiple regression models using robust standard errors, adjusting for age, sex and BMI with LVEDV and LV mass indexed to BSA and VO_2peak_ as outcomes and sporting disciplines as predictor. LVEDV and LV mass values were compared to previously published reference values.^16^ Statistical comparison of correlation coefficients was performed in line with the method described by Hittner et al.^17^ To assess whether between-centre differences in measurements might have influenced the results we performed sensitivity analyses excluding the Norwegian cohort of endurance athletes. We compared the indexation methods for LVEDV and LV mass in differentiating between athletes and DCM patients using area under the curve (AUC) statistics and compared AUCs using DeLong’s test. For statistical analyses R version 4.4.0 (2024-04-24) (R Foundation for Statistical Computing, Vienna, Austria) was used together with packages *ggplot2, cocor, ggpmisc, ggpubr, estimatr and pROC.*

## Results

A total of 85 athletes (52 male; 70 from the COSMO-S cohort and 15 from the Norwegian cohort) were included in the study, whereof 52 athletes practiced in endurance sports and 22 athletes in mixed sports, while only 9 athletes practiced power sports and 2 athletes practiced technical sports (*Table 1*). Mean age was 28 ± 8 years without significant differences across sports (*P* = 0.10). Significant differences across sports were observed for sex (*P* = 0.002), body mass index (*P* = 0.032), height (*P* = 0.018) and relative VO_2peak_ (*P* = 0.004) (*Table 1*). The cohort included more recreational (*n* = 55) than elite athletes (*n* = 31), and the recreational athletes had a significantly lower VO_2peak_ than the elite athletes (41.0 vs. 58.1 mL/kg/min, *P* < 0.001), with the elite endurance athletes having the highest VO_2peak_ with a mean of 63.7 mL/kg/min. Echocardiographic measures are shown in *Table 2*. Significant differences across sports were observed for LVEDV, LVIDd and LV mass, in which endurance and mixed athletes had higher LVEDV and LVIDd, as well as higher LV mass than athletes in power and technical sports (*Table 2*). Specifically, both endurance and mixed athletes had significantly higher LVEDV compared to power and technical athletes in pairwise comparisons between individual groups (all *P* < 0.04), while there were no significant differences between endurance and mixed athletes for LVEDV (*P* = 0.90) and between power and technical athletes (*P* = 0.36). Thirty-eight athletes (45%) had LVEDV above the upper limits of normal, and 37 athletes (44%) had LV mass above upper limits of normal.^16^

**Table 1:** General characteristics of the study sample

	Endurance athletes	Mixed	Power	Technical	*P*-value
	*n* = 52	*n* = 22	*n* = 9	*n* = 2	
Age (years)	29 (9)	24 (5)	29 (8)	22 (3)	0.100
Female sex	16 (31%)	1 (4.5%)	4 (44%)	2 (100%)	
Competing level					0.300
Elite athletes	22 (42%)	7 (32%)	1 (11%)	1 (50%)	
Recreational	30 (58%)	15 (68%)	8 (89%)	1 (50%)	
Body mass (kg)	73 (11)	80 (10)	73 (11)	63 (20)	**0.032**
Height (cm)	178 (7)	183 (8)	177 (7)	172 (12)	**0.018**
BMI (kg/m^2^)	23.1 (2.6)	24.0 (2.7)	23.5 (2.6)	20.9 (4.0)	0.400
Resting heart rate (bpm)	62 (11)	63 (11)	66 (15)	70 (6)	0.599
Systolic BP (mmHg)	122 (12)	120 (7)	116 (11)	105 (21)	0.500
Diastolic BP (mmHg)	76 (9)	78 (8)	72 (7)	65 (7)	0.110
Respiratory exchange ratio	1.20 (0.10)	1.22 (0.09)	1.21 (0.10)	1.13 (0.20)	0.800
Peak heart rate (bpm)	183 (13)	179 (11)	176 (13)	177 (6)	0.200
Relative peak oxygen uptake (mL/kg/min)	51 (15)	44 (6)	38 (6)	32 (1)	**0.004**
Percentage of predicted VO_2peak_ (%)	117 (27)	94 (11)	91 (14)	80 (13)	**<0.001**

Abbreviations: BMI, body mass index; bpm, beats per minute; BP, blood pressure at rest; VO_2peak_, peak oxygen uptake.

**Table 2:** Echocardiographic measures

	Endurance athletes	Mixed	Power	Technical	*P*-value
LVEDV (mL)	147 (42)	146 (35)	113 (22)	86 (43)	**0.034**
LVIDd (mm)	53.3 (5.7)	52.6 (4.3)	48.1 (3.0)	45.5 (4.7)	**0.014**
LV EF (%)	68 (11)	72 (7)	74 (9)	65 (20)	0.130
LV GLS (%)	−19.6 (2.2)	−18.8 (1.2)	−18.3 (1.8)	−17.9 (3.9)	0.110
IVSd (mm)	8.70 (1.22)	8.73 (1.05)	8.16 (1.26)	8.10 (1.98)	0.500
LV PWd (mm)	8.68 (1.33)	8.93 (1.23)	8.34 (1.16)	7.50 (1.13)	0.230
LV mass (g)	169 (40)	170 (37)	134 (36)	115 (49)	**0.044**
LV mass/volume ratio	1.18 (0.23)	1.18 (0.16)	1.18 (0.16)	1.37 (0.12)	0.4
Mitral E/A	1.81 (0.71)	1.69 (0.41)	1.43 (0.57)	1.40 (0.71)	0.300
Lateral e'	16.4 (3.7)	17.7 (3.9)	15.7 (3.7)	17.0 (1.4)	0.600
Septal e'	11.5 (3.6)	12.5 (2.7)	10. 6 (2.7)	13.5 (2.1)	0.063
E/e'	6.1 (1.5)	6.0 (1.7)	7.1 (1.2)	6.0 (0.5)	0.200

Abbreviations: LVEDV, left ventricular end-diastolic volume; LVIDd, left ventricular internal diameter at end-diastole; LV EF, left ventricular ejection fraction; LV GLS, left ventricular global longitudinal strain; IVSd, septal thickness; LV PWd, left ventricular posterior wall thickness; LV mass/volume ratio, LV mass to end-diastolic volume ratio. E/A, ratio of early (E) to late (A) diastolic transmitral flow velocities. Lateral e′, early diastolic velocity of the mitral annulus measured at the lateral wall. Septal e′, early diastolic velocity of the mitral annulus measured at the interventricular septum. E/e′, ratio of early (E) to average (e′).

### The association between BSA, VO_2peak_, and LV volume and mass

**Figure 1:**
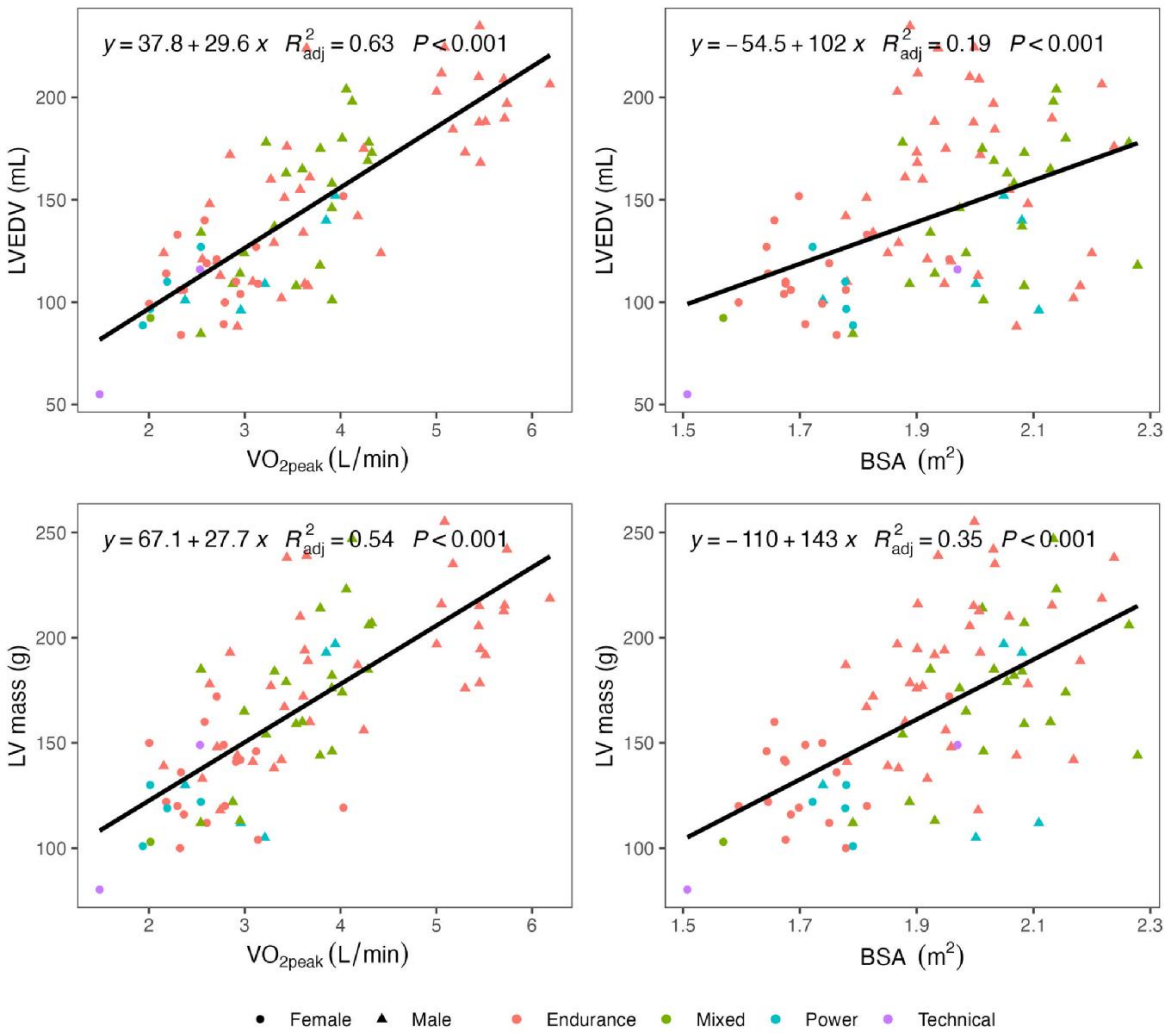
Scatterplot for relationships between VO_2peak_, BSA, LVEDV, and LV mass. Abbreviations: LVEDV, eft ventricular end-diastolic volume; VO_2peak_, peak oxygen uptake; BSA, body surface area.

In univariate regression and correlation analyses, VO_2peak_ accounted for a significantly greater proportion of the variance in LVEDV compared to BSA (R^2^ = 64% vs. R^2^ = 19%, *P* < 0.001 for the comparison of correlation coefficients). Similarly, VO_2peak_ accounted for a numerically larger proportion of the variance in LV mass compared to BSA (R^2^ = 54% vs. R^2^ = 36%); however, this difference did not reach statistical significance (*P* = 0.06). Scatter plots and corresponding linear regression results are presented in *Figure 1*.

**Figure 2:**
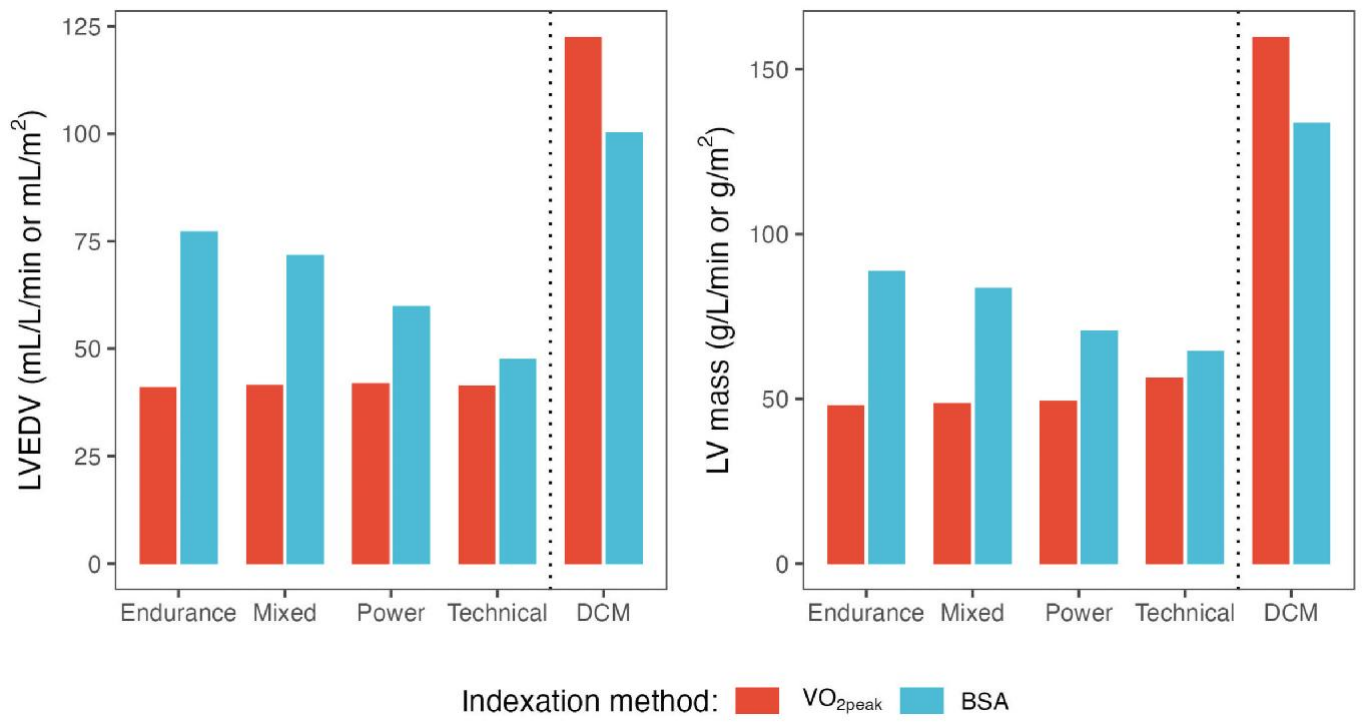
LVEDV and LV mass indexed to BSA and VO_2peak_ showing significant differences for BSA-indexed values across sporting disciplines, and no significant differences for VO_2peak_-indexed values. For reference, patients with DCM show enlarged LV size using both indexation methods. Abbreviations: DCM, dilated cardiomyopathy; LVEDV, left ventricular end-diastolic volume; VO_2peak_, peak oxygen uptake (L/min); BSA, body surface area (m^2^).

When indexing LVEDV and LV mass to BSA we observed statistically significant differences across the various sport disciplines in the full cohort (both *P* ≤ 0.019). In contrast, indexing these parameters to VO_2peak_ revealed no significant differences between groups (both *P* ≥ 0.40) (*Table 3*, *Figure 2*). In sensitivity analyses restricted to the COSMO-S cohort, performed to account for potential differences in measurements across the study centres, the results did not change considerably. Significant differences remained for unindexed LVEDV (*P* = 0.025) and LV mass (*P* = 0.040), but no significant differences were observed when indexed to VO_2peak_ (both *P* > 0.40). When restricted to the COSMO-S cohort and indexed to BSA, both LVEDV and LV mass were no longer statistically significant (*P* = 0.06 and *P* = 0.11, respectively). We also performed sensitivity analyses excluding the two technical athletes without changing results significantly. In multiple regression analyses adjusting for age, sex and BMI, there were no significant differences across sporting disciplines when indexing for VO_2peak_ for both LVEDV and LV mass as outcomes (exception for LV mass indexed to VO_2peak_ for endurance athletes vs. technical athletes). In models indexing for BSA, significant differences across sporting disciplines persisted (see Supplementary data online, *Figure S1*).

**Table 3:** LVEDV and LV mass indexed to BSA and VO_2peak_ across sporting disciplines

	Endurance athletes	Mixed	Power	Technical	*P*-value
BSA (m^2^)	1.90 (0.17)	2.02 (0.16)	1.89 (0.16)	1.74 (0.33)	**0.014**
VO_2peak_ (L/min)	3.70 (1.20)	3.52 (0.64)	2.78 (0.76)	2.01 (0.74)	**0.021**
LVEDV/BSA (mL/m^2^)	77 (20)^a,b^	72 (15)	60 (10)	48 (16)	**0.014**
LVEDV/VO_2peak_ (mL/L/min)	41 (9)	42 (7)	42 (7)	41 (6)	0.80
LV mass/BSA (g/m^2^)	89 (17)^a,b^	84 (15)	71 (16)	64 (16)	**0.019**
LV mass/VO_2peak_ (g/L/min)	48 (11)	49 (8)	49 (9)	56 (3)	0.40

^a^Significantly different from Power athletes at *P* < 0.05.

^b^Significantly different from Technical athletes at *P* < 0.05.

Abbreviations: BSA, body surface area; VO_2peak_, peak oxygen uptake; LVEDV, left ventricular end-diastolic volume.

### Comparison with DCM patients

Patients with DCM (*n* = 12) were older (mean age 65 ± 12 years), had a higher BMI, lower VO_2peak_, and a profile of generally reduced measures of systolic and diastolic function compared with the athletes (see Supplemental Material; Supplementary data online, *Table S1*). All were in New York Heart Association functional class II. The LVEDV/VO_2peak_ showed a superior AUC compared with LVEDV/BSA for differentiating athletes from DCM patients (AUC 0.99 vs. 0.74, *P* = 0.002), while LV mass/VO_2peak_ showed a small but significantly higher AUC compared with LV mass/BSA (AUC 1.00 vs. 0.96, *P* = 0.049) (see Supplementary data online, *Figure S2*). Of note, 4 of the 12 DCM patients had normal values for percentage of predicted VO_2peak_ (82 to 117% of predicted), but all had elevated LVEDV/VO_2peak_ (67 to 183 mL/L/min).

## Discussion

The study’s primary finding is that indexing LVEDV and LV mass to VO_2peak_ eliminates the differences observed across sport disciplines when using non-indexed or BSA-indexed values (Graphical Abstract). This consistency persists despite notable variations in height, body mass, BSA and relative VO_2peak_ across athletes. This result is likely due to VO_2peak_ better reflecting the metabolic demands related to body size, body composition, and training volume,^18^ making it a more comprehensive metric for evaluating cardiac adaptions in athletes. Secondly, the method improved differentiation between athletes and patients with DCM, signifying its clinical potential. Due to the small number of athletes in the power and technical groups, these findings should be considered preliminary observations requiring confirmation in larger and more balanced cohorts.

Indexing cardiac dimensions to VO_2peak_ also aligns with the geometrical similarity principle,^19^ which posits that three-dimensional parameters (e.g. LV volume) should be scaled to other cubic measures like VO₂_peak_ (L/min). This enhances the accuracy and relevance of cardiac assessments in athletic populations. Furthermore, a previous study demonstrated that the LVEDV/VO_2peak_ ratio effectively normalizes differences in LV size across body size, sex, and, to a considerable extent, age.^5^ Expanding on these findings, our data indicate that VO_2peak_-based indexing can account for differences in LVEDV and LV mass across various sport disciplines.

### Comparison with previous literature

**Figure 3:**
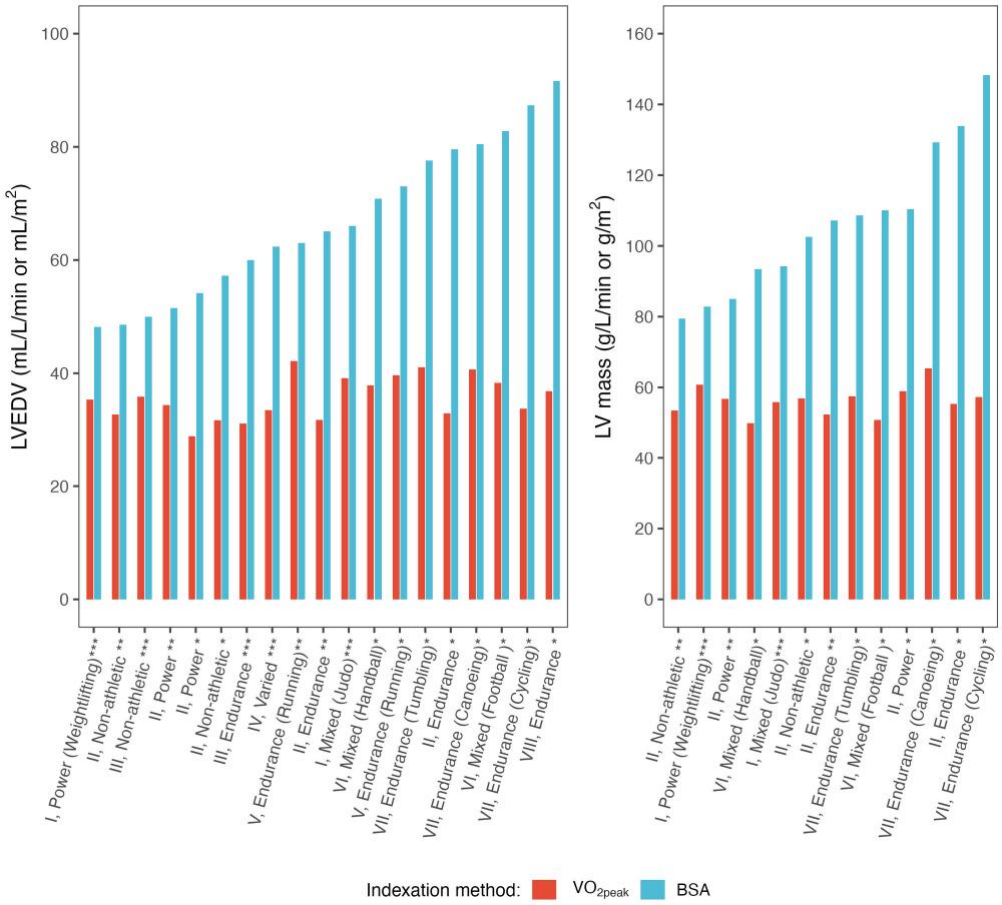
LVEDV and LV mass indexed to BSA and VO_2peak_ across various studies directly or indirectly reporting descriptive date for the four measures. Studies: I: Di Gioia et al 2024,^20^ II: Wernstedt et al. 2002,^21^ III: Rundqvist et al. 2017,^22^ IV: La Gerche et al. 2012,^23^ V: Simard et al. 2022,^24^ VI: Kandels et al. 2023,^25^ VII: Barbier et al. 2006,^26^ VIII: Sundstedt et al. 2004.^27^ Annotations: *males, **females, ***both sexes. Abbreviations: LVEDV, left ventricular end-diastolic volume; VO_2peak_, peak oxygen uptake (L/min); BSA, body surface area (m^2^).

To compare to previous findings from the literature, we compared data from multiple identified studies on both athletes and non-athletes, which provided descriptive statistics for BSA, VO_2peak_, LVEDV, and LV mass using the two indexing methods. Across these studies, LVEDV/VO_2peak_ and LV mass/VO_2peak_ ratios remained relatively stable, even in cohorts of endurance athletes, where high values for LVEDV/BSA and LV mass/BSA were reported (*Figure 3*). Although our sample size was limited, these previously published data support our findings that VO_2peak_-based indexing provides a physiologically robust approach to evaluating LV remodelling across sport types.

A large proportion of the included athletes had LV size metrics above standard echocardiographic reference values, findings which are consistent with previous studies in highly trained endurance athletes,^28^ indicating that these may underestimate normal physiological adaptations in this population.

### Implications for interpretation of the athlete's heart

These findings provide nuance to current recommendations which emphasize the importance of considering age, sex, body size, and sporting disciplines when evaluating the athlete’s heart, providing a complementary approach for interpreting LV remodelling in athletes.^2^ For measures of LV size, indexing to VO_2peak_ appears to substantially simplify interpretation, with the strongest evidence currently supporting its application to LVEDV. However, the relationship between LV mass and VO_2peak_ is more complex. Previous research indicates that while the correlation between LVEDV and VO_2peak_ remains robust across age groups, the correlation between LV mass and VO_2peak_ is strong in younger individuals but diminishes with age.^29^ This occurs because LV mass remains relatively stable across the adult lifespan, whereas LVEDV tends to decrease in proportion to declines in VO_2peak_, reflecting age-related remodelling processes in the healthy heart that involve reduced ventricular volumes but preserved systolic ejection fraction.^30^ With advancing age, both arterial blood pressure and LV wall thickness typically increase, contributing to a higher LV mass independent of metabolic demand.^31^ Thus, this age-related hypertrophic response may be related to chronically increased afterload and may, at least in part, explain observed dissociation between LV mass and VO_2peak_. This suggests that LV mass in older adults may be more strongly influenced by pressure overload than by oxygen consumption needs. Although our cohort included only young, predominantly normotensive athletes, these considerations highlight the need for caution when applying VO_2peak_-based indexing to LV mass, particularly in aging populations. It should be noted that our study population did not include older athletes and that these questions thus could not be answered in our study.

### Potential clinical implications

Indexing to VO_2peak_ allows for the evaluation of whether LV remodelling aligns with physiological norms. We show that indexing by VO_2peak_ may better differentiate athletes from patients with DCM. However, the DCM patients in our study were older than the athletes and had a mean LV ejection fraction of 30%, representing a more advanced disease stage that is generally less challenging to diagnose than early or borderline DCM phenotypes. The inclusion of a comparator group of patients with DCM adds incremental value by extending the VO_₂peak_-indexing concept from healthy individuals to a clinical population where this concept may be of special interest. However, because our DCM cohort consisted of patients with more advanced disease, generalization to earlier or borderline DCM phenotypes should be made with caution. The main diagnostic challenge in sports cardiology lies in distinguishing physiological remodelling from early or subtle cardiomyopathic changes, and future studies including such populations are warranted to explore the clinical applicability of this approach.

Although the method may have the potential to detect subtle or preclinical pathological remodelling, our data does not provide evidence for this. Incorporating VO_2peak_ into clinical assessments could enhance the precision of preparticipation screenings and routine evaluations by flagging athletes for additional diagnostics, such as cardiac magnetic resonance imaging or genetic testing. Moreover, longitudinal monitoring of VO_2peak_-indexed measures may help track disease progression or evaluate responses to interventions. While this approach is not necessarily intended to replace deconditioning as a diagnostic strategy, it could reduce the need for deconditioning in what is today thought to be ‘grey zone’ athletes by providing reference to whether cardiac remodelling is within physiologically expected limits. Thus, we believe this approach highlights the valuable information that CPET can provide in the evaluation of athletes and in other patient groups undergoing cardiac work-up. Still, further research is needed to validate its role in detecting pathology and refining personalized management strategies.

## Limitations

The number of athletes in the mixed, power and technical groups was very low, highlighting the need to replicate these findings in larger and more diverse cohorts due to the risk of a type 2 error. The low female representation in our cohort unfortunately also limited the possibility to perform sex-specific analyses and restricts the generalizability of our findings, as cardiac remodelling and VO_2peak_ responses are known to differ by sex.^32^ Additionally, the use of different exercise modalities for CPET across cohorts represents an important limitation, as VO_2peak_ is influenced by sport-specificity and ergometer type. The heterogeneity could have affected comparability of VO_2peak_ and, consequently, the indexed cardiac measures. When using bicycle ergometry, VO_2peak_ may be underestimated, particularly in older individuals, whereas this effect is less pronounced in younger, well-trained populations. The lack of a standardizes test protocol across cohorts could therefore have introduced some degree of systematic bias, although sensitivity analyses omitting the Norwegian cohort from analyses did not significantly impact the results. The athletes from COSMO-S were included after SARS-CoV-2 infection, and although all were examined at least three months after testing positive, residual or long-term cardiopulmonary effects from prior infection cannot be fully excluded. The use of different echocardiographic systems (Philips and GE) might have led to some measurement variability between cohorts, although such, if any, differences are expected to be small given the results from sensitivity analyses restricted to one cohort. Finally, performing echocardiography and CPET sequentially as in this study may limit direct structure-function correlations compared to simultaneous approaches.^33^

## Conclusions

Indexing LV volumes and mass to VO_2peak_ reduces the differences observed across sport disciplines when using non-indexed or BSA-indexed values and offers a promising method for integrating body size *and* training status into a single measure. In our evaluation of athletes and DCM patients, this approach improved the differentiation between physiological and pathological LV enlargement. Nonetheless, these results are exploratory and should be validated in further studies with broader representation across sporting disciplines and disease severities.

## Supplementary Material

qyaf138_Supplementary_Data

## Data Availability

The data underlying this article cannot be shared publicly due to data protection regulations. The data will be shared on a reasonable request to the corresponding author.

## Lead author biography

Dr. Jana Schellenberg is a cardiologist and specialist in internal medicine with additional qualifications in sports and emergency medicine. Her expertise spans cardiac imaging, diagnostics, and preventive cardiology. Her research focuses on myocardial function, long-term cardiovascular effects of COVID-19 in athletes (COSMO Study) and post-COVID patients (EPILOC Study), as well as exercise-based prevention of cancer therapy–related cardiac dysfunction (CHiB Study). She serves on the Sports Cardiology Nucleus of the German Society of Cardiology, as speaker of the Sports Cardiology Group within the German Association of Sports Medicine, and as Young Ambassador for the European Association of Preventive Cardiology.
