# Supplementary material for: Physiological assessment of left ventricular size indexed by peak oxygen uptake across sporting disciplines
Source: Eur Heart J Imaging Methods Pract. 2025 Oct 30;3(4):qyaf138. doi: 10.1093/ehjimp/qyaf138 (PMC12619062; doi:10.1093/ehjimp/qyaf138)
Supplement: qyaf138_Supplementary_Data [file qyaf138_supplementary_data.pdf]

SUPPLEMENTAL MATERIAL:

Physiological assessment of left ventricular size indexed by peak oxygen uptake across sporting disciplines

Jana Schellenberg[1], Lynn Matits[1, 2], Johannes Kersten[1], Daniel Alexander Bizjak[1], Johannes Kirsten[1], Thomas Fremo[3], Arnt Erik Tjønna[3], Knut Skovereng[4], Øyvind Sandbakk[5], Inger-Lise Aamot Aksetøy[3,6,7], Knut Asbjørn Rise Langlo[8,11], Håvard Dalen[3,9,10], Jon Magne Letnes[3,10]*

[1] Sports and Rehabilitation Medicine, University Hospital Ulm, Leimgrubenweg 14, 89075 Ulm, Germany

[2] Clinical & Biological Psychology, Institute of Psychology and Education, Ulm University, 89075 Ulm, Germany

[3] Department of Circulation and Medical Imaging, Norwegian University of Science and Technology, Trondheim, Norway

[4] Centre for Elite Sports Research, Department of Neuromedicine and Movement Science, Norwegian University of Science and Technology, 7491, Trondheim, Norway.

[5] School of Sport Science, UiT The Artic University of Norway, Tromsø, Norway

[6] National Advisory Unit on Exercise Training as Medicine for Cardiopulmonary Conditions, Trondheim, Norway

[7] Clinic of Rehabilitation, St. Olavs University Hospital, Trondheim, Norway

[8] Department of Nephrology, Clinic of Medicine, St. Olavs Hospital, Trondheim University Hospital, Prinsesse Kristinas gate 3, 7030 Trondheim, Norway

[9] Department of Medicine, Levanger Hospital, Nord-Trøndelag Hospital Trust, Kirkegata 2, 7600 Levanger, Norway

[10] Clinic of Cardiology, St. Olavs University Hospital, Prinsesse Kristinas gate 3, 7030 Trondheim, Norway

[11] Department of Clinical and Molecular Medicine, Norwegian University of Science and Technology, Trondheim, Norway

***Corresponding author:**

Jon Magne Letnes[3,10] MD PhD

E-mail: jon.m.letnes@ntnu.no

Phone: +47 90 51 31 93

Supplemental Table 1. Comparison of general characteristics, echocardiographic measures, and indexed measures between patients with dilated cardiomyopathy, all athletes, and elite endurance athletes.

	**All athletes,** n=85	**DCM,** n=12	**p-value**	**Elite endurance athletes,** n=22	**DCM,** n=12	**p-value**
Age (years)	28 (8)	65 (12)	<0.001	24 (5)	65 (12)	<0.001
Female sex	23 (27%)	2 (17%)	<0.001	5 (23%)	2 (17%)	<0.001
Body mass (kg)	75 (11)	87 (16)	0.019	74 (10)	87 (16)	0.014
Height (cm)	179 (8)	180 (7)	0.7	179 (7)	180 (7)	0.8
BMI (kg/m^2)	23.3 (2.6)	27.0 (4.2)	0.011	22.9 (1.7)	27.0 (4.2)	0.006
Resting heart rate (bpm)	63 (11)	66 (12)	0.4	57 (11)	66 (12)	0.039
Systolic BP (mmHg)	120 (11)	119 (17)	0.8	122 (8)	119 (17)	0.6
Diastolic BP (mmHg)	76 (9)	74 (14)	0.7	72 (7)	74 (14)	0.6
Respiratory exchange ratio	1.21 (0.10)	1.06 (0.07)	<0.001	1.15 (0.10)	1.06 (0.07)	0.005
Peak heart rate (bpm)	181 (12)	128 (18)	<0.001	190 (11)	128 (18)	<0.001
Peak oxygen uptake (mL/kg/min)	47 (13)	21 (5)	<0.001	64 (12)	21 (5)	<0.001
Percentage of predicted VO_{2peak} (%)	107 (25)	75 (16)	<0.001	137 (22)	75 (16)	<0.001
LVEDV (mL)	142 (40)	207 (75)	0.012	173 (39)	207 (75)	0.2
LVIDd (mm)	52 (5)	65 (10)	0.002	57 (5)	65 (10)	0.031
LV EF (%)	70 (10)	30 (11)	<0.001	60 (9)	30 (11)	<0.001
IVSd (mm)	8.63 (1.19)	9.79 (1.37)	0.015	8.81 (1.04)	9.79 (1.37)	0.044
LV PWd (mm)	8.68 (1.29)	9.12 (2.73)	0.6	8.87 (1.52)	9.12 (2.73)	0.8
LV mass (g)	164 (41)	275 (50)	<0.001	191 (36)	275 (50)	<0.001
LV mass/volume ratio	1.19 (0.21)	1.34 (0.40)	0.2	1.11 (0.17)	1.34 (0.40)	0.085
Mitral E/A	1.73 (0.63)	2.03 (2.10)	0.7	2.23 (0.78)	2.03 (2.10)	0.8
Lateral e'	16.7 (3.7)	6.5 (3.4)	<0.001	17.7 (3.3)	6.5 (3.4)	<0.001
Septal e'	11.7 (3.3)	4.7 (1.5)	<0.001	10.9 (2.1)	4.7 (1.5)	<0.001
E/e'	6.15 (1.52)	14.31 (7.11)	0.002	5.3 (1.0)	14.3 (7.1)	0.001
BSA (m^2)	1.93 (0.17)	2.06 (0.20)	0.039	1.92 (0.16)	2.06 (0.20)	0.042
Peak oxygen uptake (L/min)	3.52 (1.09)	1.87 (0.59)	<0.001	4.70 (1.13)	1.87 (0.59)	<0.001
LVEDV/BSA	73 (19)	100 (36)	0.026	90 (18)	100 (36)	0.4
LVEDV/VO_2	41 (8)	123 (66)	0.001	38 (6)	123 (66)	<0.001
LV mass/BSA	85 (18)	134 (32)	<0.001	100 (14)	134 (32)	0.005
LV mass/VO_2	49 (10)	160 (62)	<0.001	42 (9)	160 (62)	<0.001

[1] Mean (SD); n (%)
[2] Two Sample t-test; Fisher's exact test

Abbreviations: DCM=Dilated Cardiomyopathy, BMI=Body Mass Index, Bpm=Beat per minute, BP=Blood Pressure at rest, LVEDV=Left ventricular end-diastolic volume, LVIDd=Left ventricular internal diameter at end-diastole, LV EF=Left ventricular ejection fraction, IVSd=Septal thickness, LV PWd=Left ventricular posterior wall thickness, LV mass/volume ratio=LV mass to end-diastolic volume ratio. E/A=Ratio of early (E) to late (A) diastolic transmitral flow velocities. Lateral

e`=Early diastolic velocity of the mitral annulus measured at the lateral wall. Septal e`=Early diastolic velocity of the mitral annulus measured at the interventricular septum. E/e`=Ratio of early (E) to average (e`), BSA=Body surface area, VO_{2peak}=Peak oxygen uptake.

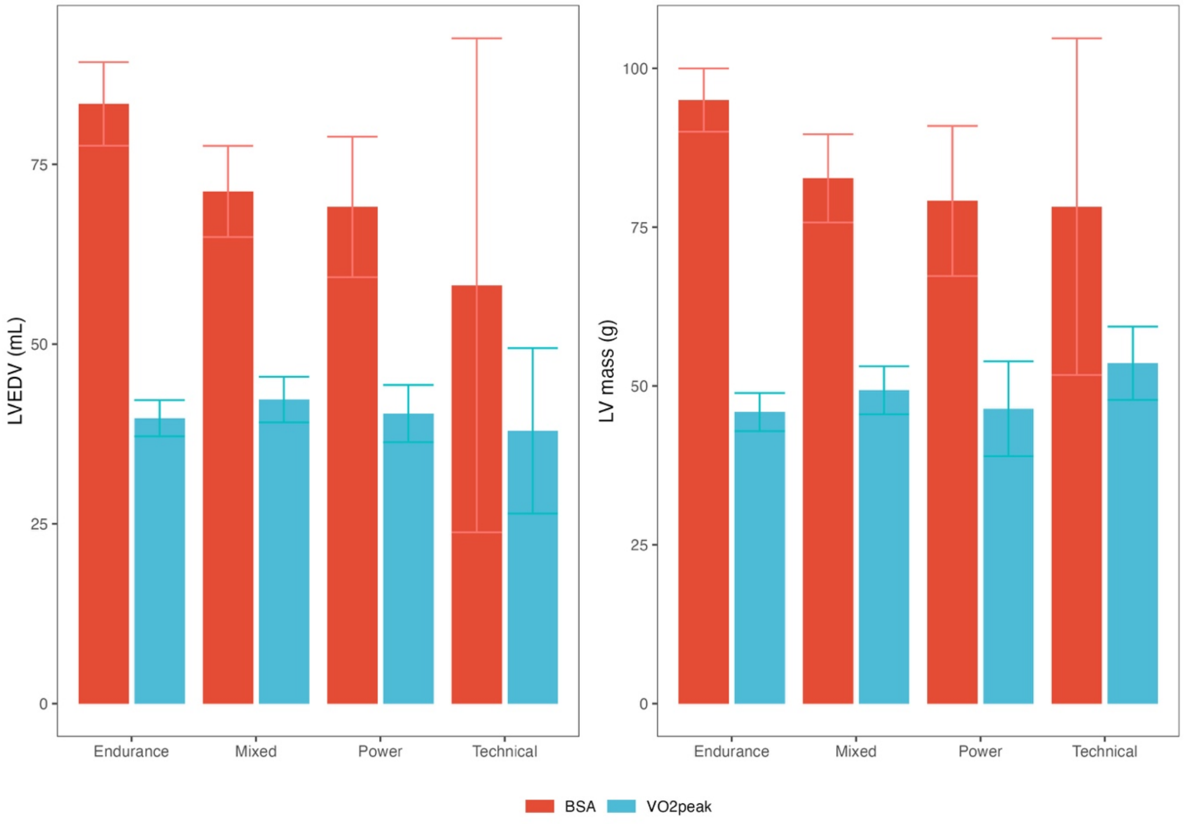

Supplemental Figure 1. Estimated means with 95% confidence intervals (error bars) from multiple regression models adjusted for age, sex, and body mass index for left ventricular end-diastolic volume (LVEDV) and left ventricular (LV) mass, indexed to body surface area (BSA, m^2) and absolute peak oxygen uptake (VO_{2peak}, L/min).

Significant differences were found between the endurance athletes and both mixed and power athletes, but not for technical athletes, for LVEDV indexed to BSA. There were no significant groupwise comparisons when LVEDV was indexed to $VO_{2peak.}$ For LV mass indexed to BSA, significant differences were found between endurance athletes and both mixed and power athletes, respectively, but no other comparisons were significant. For LV mass indexed to VO_{2peak}, the only significant groupwise difference was between endurance and technical athletes.

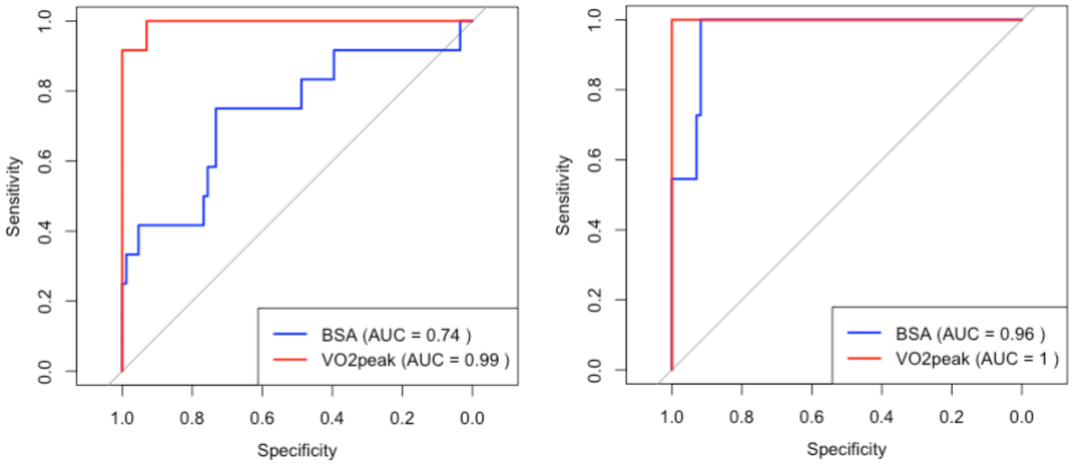

Supplemental Figure 2. Receiver operating characteristics curves for differentiating athletes from patients with dilated cardiomyopathy using left ventricular end-diastolic volume (left) and left ventricular mass (right), indexed to body surface area (BSA) and peak oxygen uptake (VO_{2peak}).
